# Synthesis of Polymer Sodium Alginate–Red Mud Adsorbent and Its Application in the Removal of Low-Concentration Fluoride

**DOI:** 10.3390/polym17060826

**Published:** 2025-03-20

**Authors:** Jiahao Wang, Huali Zhang, Nenghao Wang, Han Mo, Zhen Yang, Yangyang Dong, Qingwei Liu, Xiao Huang, Baoyi Han

**Affiliations:** School of Chemistry and Environmental Engineering, Wuhan Institute of Technology, Wuhan 430205, China; jiahao_wang211@163.com (J.W.); hang050408@163.com (N.W.); 13886394087@163.com (H.M.); 15623959920@139.com (Z.Y.); 19107151022@163.com (Y.D.); lqw041212@163.com (Q.L.); smhx2012@126.com (X.H.); 15307134616@163.com (B.H.)

**Keywords:** sodium alginate, red mud, fluoride removal, adsorbents, mechanisms

## Abstract

The sustainable management of industrial byproducts represents a critical challenge for the aluminum industry. This study developed a cost-effective adsorbent (SA@RM) derived from sodium alginate and red mud for fluoride removal, addressing both solid waste utilization and water purification needs. Systematic adsorption experiments revealed optimal performance under conditions of 15 g/L dosage and pH 5, achieving adsorption equilibrium within 40 min for initial fluoride concentrations of 11.7 mg/L. Notably, the adsorbent demonstrated exceptional cyclic stability, maintaining 54.8% adsorption capacity through three regeneration cycles. The adsorption process followed the Langmuir isotherm model (R^2^ = 0.994) and pseudo-second-order kinetics (R^2^ = 0.975), indicating monolayer chemisorption as the dominant mechanism. Advanced characterization techniques (SEM-EDS, FT-IR, XPS) elucidated three main mechanisms: fluoride complexation with aluminum oxides, ligand exchange with surface hydroxyl groups, and ion exchange with chloride species. This material achieves 92% fluoride removal while valorizing industrial waste, reducing adsorbent production costs by 60–70% compared to conventional materials. The detailed mechanism analysis provides fundamental insights for designing waste-derived adsorbents, offering a practical solution for sustainable industrial development and water treatment applications.

## 1. Introduction

With the rapid growth of industries such as manufacturing and pharmaceuticals, wastewater treatment has become extremely challenging, especially the removal of fluoride from wastewater. Although fluoride is an essential trace element and is beneficial to the production and maintenance of healthy bones and teeth, excessive fluoride intake will lead to fluorosis, bringing about skeletal and neurological diseases. Fluoride is widely present in solid waste, which leaks into rivers, soil, groundwater, and other water sources due to inappropriate disposal and improper storage. Long-term exposure to low concentrations of fluorides poses a serious threat to the growth and survival of organisms, especially aquatic animals. At the same time, fluoride also affects human survival and growth. The World Health Organization (WHO) guidelines state that the upper limit of fluoride concentration in drinking water is 1.5 mg/L [1]. However, high concentrations of fluoride can be detected in drinking water in many parts of the world, such as India, Central Africa, and South America. Therefore, the search for efficient removal methods of elemental fluorine is of important significance for environmental protection and human health.

Fluoride removal methods mainly include adsorption [2]; electro-coagulation [3]; membranes [4]; ion exchange [5]; chemical precipitation, and coagulation, and sedimentation [6]; etc. Although membrane and ion exchange methods are efficient, they are extremely costly. Chemical precipitation and coagulation precipitation can effectively remove high concentrations of fluoride, but they are not very effective in removing low concentrations of fluoride. The adsorption method is widely used due to its effectiveness, environmental friendliness, and cost-efficiency. Currently, the commonly used adsorbents for removing fluoride include adsorbents containing activated aluminum, natural minerals, and biochar [7]. Adsorbents containing activated aluminum are excellent for fluorine adsorption but have poor cycling stability. Biochar materials have extremely high cyclic stability and excellent adsorption capacity but also suffer from high production costs and separation difficulties [8]. Therefore, finding an adsorbent material that is low-cost, highly efficient, and easy to recycle has become a trend.

Red mud is a porous solid waste that is discharged in large quantities during the production of alumina from bauxite. The main issue related to red mud is the continuously increasing production that needs to be stored. It is estimated that the current global stockpile exceeds 2.7 billion tons and is increasing at a rate of 120 million tons per year [9], highlighting the severity of this problem. Therefore, it is currently necessary to broaden the utilization pathways of red mud to alleviate this issue. Its specific surface area can reach up to 180 m^2^/g, which gives it the prerequisite conditions to become an adsorbent material [10]. Moreover, research on red mud as an adsorbent material mainly focuses on the removal of heavy metal ions from water bodies. The removal of non-metal ions primarily concentrates on arsenic and phosphorus, with a lack of studies on fluoride adsorption. It can be used for efficient fluorine adsorption due to its porous surface and highly reactive alumina [11]. However, its intrinsic adsorption capacity is low, and it usually needs to be activated by high-temperature roasting and chemical modification to enhance its adsorption ability [12]. Degui Li and Bing He et al. chemically modified red mud with different concentrations of FeCl_3_, polysilicic acid, and citric acid to achieve an adsorption capacity of 0.94 mg/g [13]. Ning Wei, Zhaokun Luan, et al. chemically modified red mud with AlCl_3_ modification, which greatly improved the adsorption performance of red mud [14]. The results indicate that modified red mud significantly improved fluoride removal efficiency, demonstrating that Al plays an important role in fluoride removal. However, due to the viscous state of red mud after adsorption, there is a problem with separation after adsorption.

Most of the current adsorbents are in powder and fiber form. Although they have a large specific surface area, they present challenges in separation during application [15]. In order to give full play to the adsorption capacity of solid waste red mud and improve its separation performance, many researchers began to study porous and bulk adsorbents. Wenfei Li, Zhe Wang, et al. [16]. successfully found that a cylindrical structure adsorbent of SA-encapsulated activated alumina exhibits a high adsorption capacity of 5.09 mg/g (fluorine solution concentration of 20 mg/L). Furthermore, their characterization and adsorption experiments demonstrate that the adsorption mechanism is mainly ion exchange. However, the preparation of activated alumina is relatively complicated, and its price is higher than that of red mud [17]. Xiaotong Bian, Zhaofu Qiu, et al. fabricated a spherical adsorbent of SA@LHMS [18], and the separation performance exhibited slight improvement. However, the adsorption time was considerably prolonged, and the adsorption capacity decreased due to the active substance embedded in the SA. The adsorption equilibrium time was extended to 12 h. Moreover, researchers have conducted limited studies on the adsorption mechanisms of these types of adsorbents [19].

This study addresses three challenges in fluoride removal technology: (1) the limited adsorption capacity of unmodified red mud, (2) practical separation difficulties of powdered adsorbents, and (3) insufficient mechanistic understanding of composite adsorbents. To bridge these gaps, we aim to (i) develop a low-cost, easily separable spherical adsorbent (SA@RM) by integrating chemically modified red mud with sodium alginate, (ii) systematically evaluate its fluoride removal performance under varying operational conditions (pH, dosage, regeneration cycles), and (iii) elucidate the adsorption mechanisms through various characterization methods. Leveraging red mud’s inherent aluminum reactivity while enhancing its structural stability simultaneously valorizes industrial solid waste and enables efficient water purification. The mechanistic insights derived from this study provide a foundational framework for optimizing waste-derived adsorbents, offering scalable solutions for sustainable industrial practices and water treatment systems.

## 2. Experimental

### 2.1. Materials

Red mud came from Shandong Aluminum Company (Jinan, China) Sodium alginate (SA) was purchased from Aladdin Biochemical Science and Technology Co., Ltd. (Shanghai, China). Sodium fluoride (NaF) was purchased from Aladdin Biochemical Technology Company Limited (Shanghai, China). NaOH and HCl were purchased from Aladdin Biochemical Technology Company Limited (Shanghai, China). The water used in the laboratory was deionized, and fluoridated wastewater was prepared in the laboratory.

### 2.2. Preparation of Adsorbents

#### 2.2.1. Modification and Activation of Red Mud

**Figure 1:**
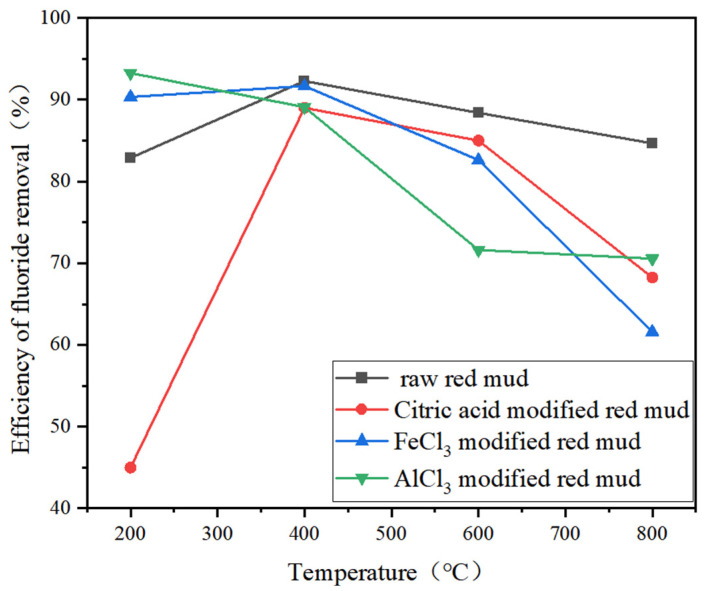
Effect of temperature on efficiency of fluoride removal by raw red mud (initial concentration 11.7 mg/L, dosage 20 g/L, pH = 5).

The main chemical composition of the red mud used in the laboratory is shown in Table 1. At room temperature, the red mud was sieved and then dried at 55 °C for 24 h. Next, 100 g samples were added into 150 mL of solution, (5wt% FeCl_3_, citric acid, AlCl_3_ solution,), stirred thoroughly, and filtered after soaking at 25 °C for 24 h. Then, the activated red mud was dried for 48 h and calcined at 200 °C, 400 °C, 600 °C, and 800 °C for 2 h. The adsorption experiment was carried out in 100 mL of fluorine-containing wastewater with a concentration of 11.7 mg/L using the above red mud under the conditions of 2 g dosage at pH = 4–5. The adsorption results are shown in Figure 1. Although the 200 °C AlCl_3_-modified red mud was the most effective in removing fluorine, it was difficult to form a spherical shape due to the fact that Al^3+^ can also cross-link with sodium alginate. Therefore, unmodified red mud roasted at 400 °C was chosen as the raw material for adsorbent preparation.

#### 2.2.2. Preparation of SA@RM

The red mud roasted at 400 °C for 2 h was added to the deionized water at a dosage of 80 g/L and stirred thoroughly. Then, 1.5% SA was added and stirred thoroughly at room temperature to dissolve it. Afterward, the mixture was added drop by drop to the CaCl_2_ solution with a mass fraction of 2% to form SA@RM spheres using a rubber-tipped burette. After 12 h of crosslinking, the spheres were washed with deionized water and freeze-dried for 12 h. SA@RM can be used for fluoride removal without further treatment.


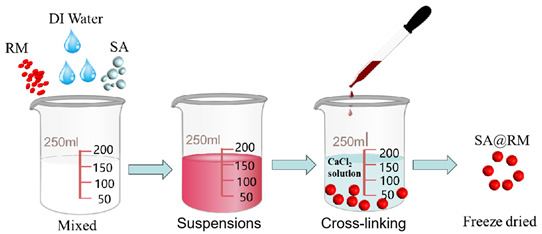


### 2.3. Characterization of Materials

A scanning electron microscope (SEM, Hitachi, Tokyo, Japan) examined the surface morphology of the materials, and the elements on the material were determined by energy dispersive spectroscopy (EDS, Oxford, Xplore 30, UK) with an accelerating voltage of 20 keV. Fourier transform–infrared spectroscopy (FT-IR) was performed using a Nicolet iS50 (Thermo Scientific, Waltham, MA, USA) with an optical range of 400–4000 cm^−1^. Chemical composition and metal valence were analyzed by X-ray photoelectron spectroscopy (XPS, ESCALABII, Thermo Fisher, Waltham, MA, USA) using the binding energy of C1s (284.8 eV) as a control standard and Al-Ka as the X-ray source (1486.6 eV photons).

### 2.4. Experimental Methods

The adsorption of the fluorine solution was carried out at dosages of 5, 10, 15, 20, and 25 g·L^−1^ to investigate the effect of dosage on adsorption. The effect of pH on adsorption was investigated by controlling the pH of the solution in the range of 3–9 at 15 g·L^−1^. Cyclic regeneration experiments were performed in 0.5 mol·L^−1^ sodium hydroxide solution, and the regenerated material continued to be used for fluoride removal [20].

Adsorption kinetic experiments were carried out at an initial fluorine concentration of 11.7 mg·L^−1^ with an adsorbent dosage of 15 g·L^−1^. The adsorption kinetics of the adsorbent were determined by the following method [21].

(1) $$q_{t}=\frac{{(C}_{0}-C_{t})V}{m}$$

The pseudo-first-order kinetic model is shown below:

(2) $$q_{t}=q_{e}(1-e^{-k_{1}t})$$

The pseudo-second-order kinetic model is shown below:

q_t_ = k_2_q_e_^2^t/(1 + k_2_q_e_t) (3)

where c_0_ and c_t_ (mg·L^−1^) are the initial concentration of F^−^ in aqueous solution and the concentration at time t, respectively; V is the volume of the solution (L); and m is the mass of the SA@RM adsorbent (g). q_t_ is the adsorption capacity (mg·g^−1^), q_e_ is the adsorption capacity at equilibrium (mg·g^−1^), t is the adsorption time (min), k_1_ is the pseudo-first-order kinetic modeling rate constant (min^−1^), and k_2_ is the pseudo-second-order kinetic modeling rate constant (g·mg^−1^·min^−1^) [22].

Adsorption isothermal experiments were performed in fluorine concentrations of 5–25 mg·L^−1^. The fluorine content after adsorption was determined using the fluoride ion selective electrode method. Adsorption isotherms were fitted using the Langmuir and Freundlich mathematical equations.

Langmuir isothermal model:

q_e_ = K_L_q_m_C_e_/(1 + C_e_) (4)

Freundlich isothermal model:

q_e_ = K_F_C_e_^(−n)^ (5)

where q_e_ is the adsorption capacity at equilibrium (mg·g^−1^), C_e_ is the equilibrium concentration (mg·L^−1^), q_m_ is the maximum adsorption capacity of SA@RM for F^−^ (mg·g^−1^), K_L_ (L·mg^−1^) and K_F_ (L·mg^−1^) are the Langmuir and Freundlich isothermal constants, and n is the heterogeneity factor [23].

## 3. Results and Discussion

### 3.1. Effect of Dosage on Fluoride Removal

**Figure 2:**
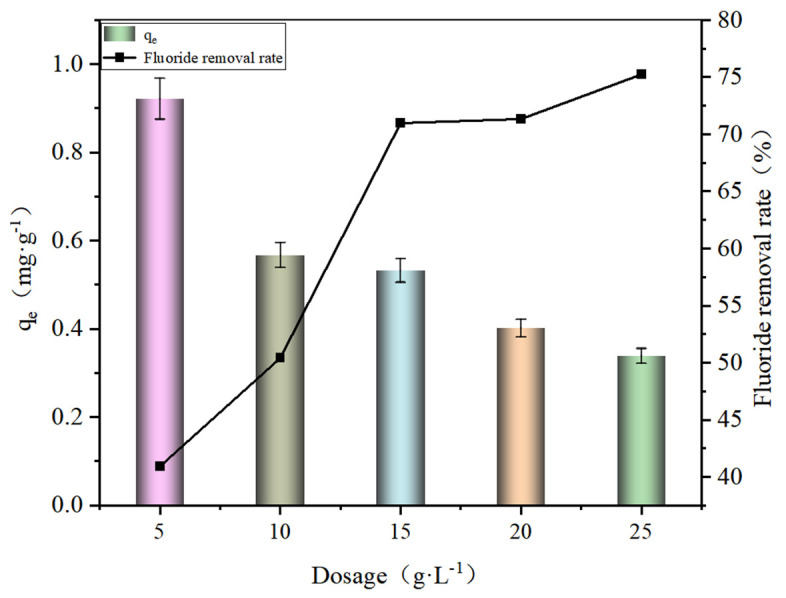
Effect of SA@RM dosage on F^−^ adsorption performance (initial concentration 11.7 mg/L, pH = 5).

The effect of SA@RM dosage on F^−^ adsorption performance is shown in Figure 2.

As can be seen from Figure 2, the F^−^ removal ratio showed an increasing trend with the increase in SA@RM dosage. When the dosage was 25 g·L^−1^, the removal rate of fluorine was 75.24%, which was much higher than that of 40.91% when the dosage was 5 g·L^−1^. In contrast the adsorption capacity showed a decreasing trend, with the adsorption capacity decreasing from 0.922 to 0.339 mg·g^−1^. When the dosage is low, the active sites of the adsorbent are sufficiently bound to fluorine. Therefore, it exhibits a high adsorption capacity. However, due to the limited active sites, the removal efficiency of fluorine is low. As the dosage increased, a large number of adsorption sites increased, leading to an increase in the removal ratio of fluorine. However, the adsorption capacity wass reduced due to the large amount of adsorbent resulting in lower access to fluorine for each adsorbent. Combining the F^−^ removal ratio and the adsorption capacity, the dosage of SA@RM was selected as 15 g·L^−1^.

### 3.2. Effect of pH on Adsorption Effect

**Figure 3:**
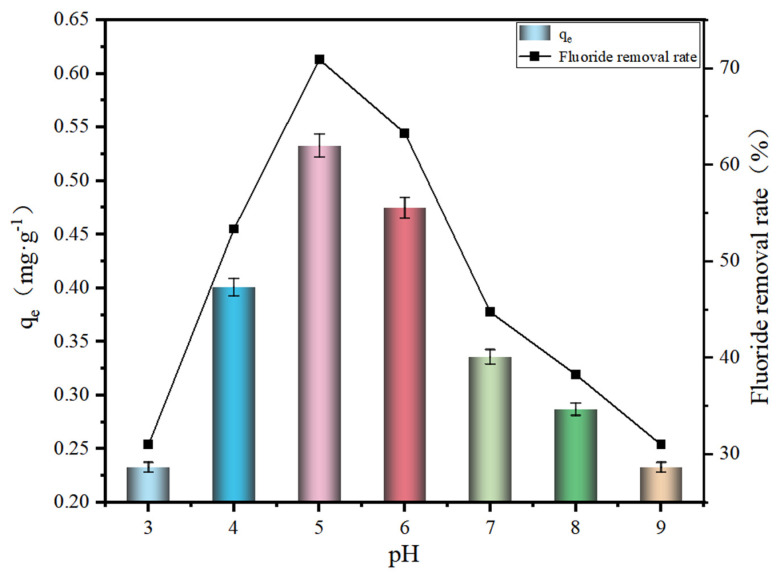
Effect of pH on effectiveness of fluoride removal (initial concentration 11.7 mg/L, dosage 15 g/L).

The pH effect on the adsorption performance is shown in Figure 3. The adsorption capacity of SA@RM showed a trend of increasing and then decreasing with the increase in pH value. The maximum adsorption capacity of SA@RM was up to 0.533 mg·g^−1^ at pH = 5. The fluorine removal ratio showed the same trend as the adsorption capacity with the pH value increasing. Due to weakly acidic conditions, the adsorption active site of SA@RM is protonated by H^+^, making its adsorption active site positively charged, and electrostatic attraction promotes F binding with the active site [24]. Furthermore, the strong electronegativity of fluorine likely facilitates hydrogen bonding with surface hydroxyl groups, thereby enhancing adsorption performance.

M-OH + H^+^→M-OH_2_^+^ (6)

M-OH_2_^+^ + F^−^→M-F + H_2_O (7)

M-OH + F^−^→M-O-H···F^−^ (8)

M stands for the surface of SA@RM.


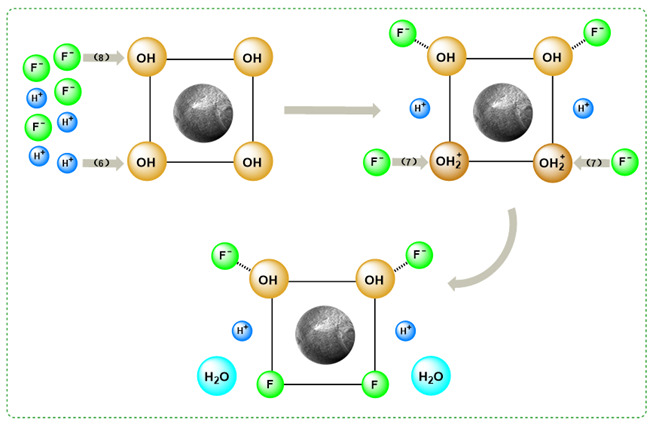


When the acidity is too strong, a large number of free H^+^ active species in the solution combine with F^−^ to form HF and HF_2_^+^, thus reducing the activity of F^−^ in the solution. At the same time, the positively charged HF_2_^+^ also produces electrostatic repulsion with the adsorption active sites, preventing fluoride from adsorbing on the surface of the SA@RM and thus leading to a decrease in the adsorption capacity [25,26]. On the other hand, under alkaline conditions, the active sites on the SA@RM surface carry a negative charge, creating electrostatic repulsion with F^−^, making it difficult for them to come into contact. In addition, OH^−^ competes with F^−^ for the adsorption sites, leading to a decrease in the adsorption capacity of the adsorbent.

### 3.3. Adsorption Kinetics and Isotherms

**Figure 4:**
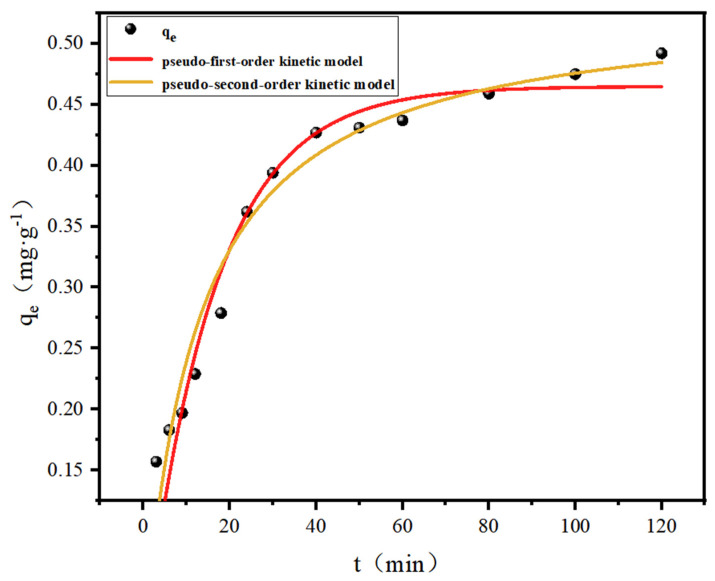
Adsorption kinetic curve (initial concentration 11.7 mg/L, dosage 15 g/L, pH = 5).

The fluoride adsorption kinetics, as illustrated in Figure 4, demonstrate a rapid initial uptake phase, with 85% of the maximum adsorption capacity (0.534 mg·g^−1^) achieved within the first 40 min. Equilibrium was subsequently attained, as evidenced by the plateau in adsorption capacity beyond this timeframe. Kinetic analysis using pseudo-first-order and pseudo-second-order models (Table 2) revealed a superior correlation with the pseudo-second-order model (R^2^ = 0.975 vs. 0.942 for the pseudo-first-order). This alignment indicates that the adsorption rate is governed by both temporal factors and adsorbate concentration gradients, characteristic of chemisorption mechanisms involving covalent interactions or electron sharing between fluoride ions and active sites (e.g., Al-OH groups). The dominance of chemical binding pathways is further supported by the observed agreement with Langmuir monolayer adsorption behavior.

**Figure 5:**
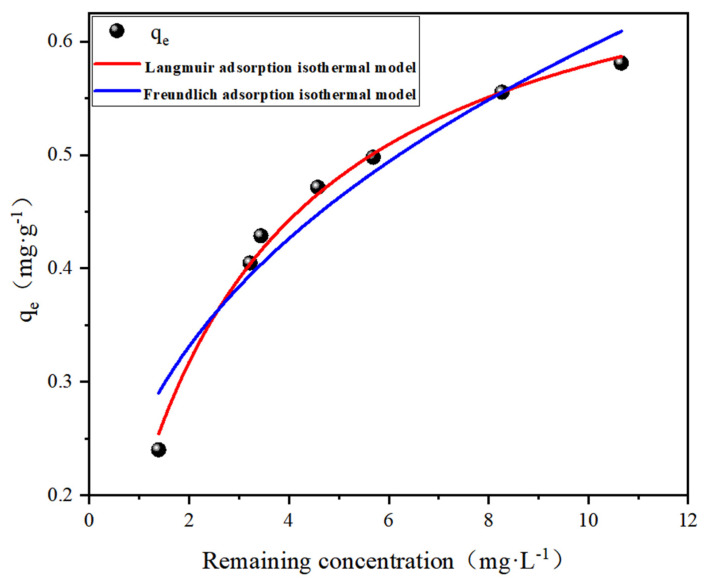
Adsorption isothermal model (dosage 20 g/L, pH = 5).

The equilibrium adsorption behavior was analyzed through nonlinear regression of the Freundlich and Langmuir isotherm models (Figure 5), revealing a concentration-dependent adsorption profile. The equilibrium adsorption capacity increased sharply at low fluoride concentrations (<15 mg·L^−1^) due to abundant available active sites (e.g., Al-OH, -COOH) on SA@RM, reaching 95% saturation at 25 mg·L^−1^. This indicates the complete occupation of monolayer binding sites, consistent with Langmuir’s assumption of homogeneous surface adsorption energy (R^2^ = 0.994 vs. Freundlich R^2^ = 0.926, Table 3). The superior Langmuir fit suggests chemisorption dominance, likely through specific interactions such as ligand exchange—fluoride substitution with hydroxyl groups—and surface complexation—the formation of Al-F bonds evidenced by XPS. The Freundlich model’s poorer fit further rejects multilayer physisorption mechanisms, confirming the monolayer-limited nature of fluoride uptake [27]. These findings align with the pseudo-second-order kinetic results, collectively demonstrating that fluoride removal primarily occurs through site-specific chemical reactions rather than physical accumulation.

### 3.4. Regeneration of Adsorbents

**Figure 6:**
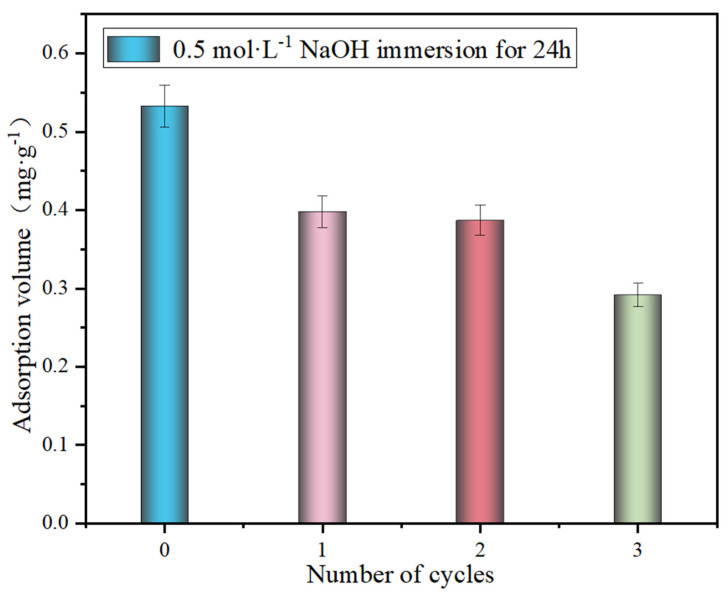
Regeneration effect of adsorbent (initial concentration 11.7 mg/L, dosage 15 g/L, pH = 5).

Figure 6 demonstrates the adsorption performance of SA@RM after regeneration in 0.5 mol·L^−1^ NaOH. It can be seen that the adsorption capacity was reduced to 0.398 mg·g^−1^ after one regeneration and 0.292 mg·g^−1^ after three regenerations. The adsorption capacity was still able to reach the initial 54.8% after three cycles, indicating the good cyclic stability of SA@RM. Moreover, the red mud embedded in the SA will not fall off, preventing secondary pollution. Therefore, SA@RM has good economic and environmental values.

### 3.5. Exploration of the Adsorption Mechanism

#### 3.5.1. BET and SEM-EDS Analysis

**Figure 7:**
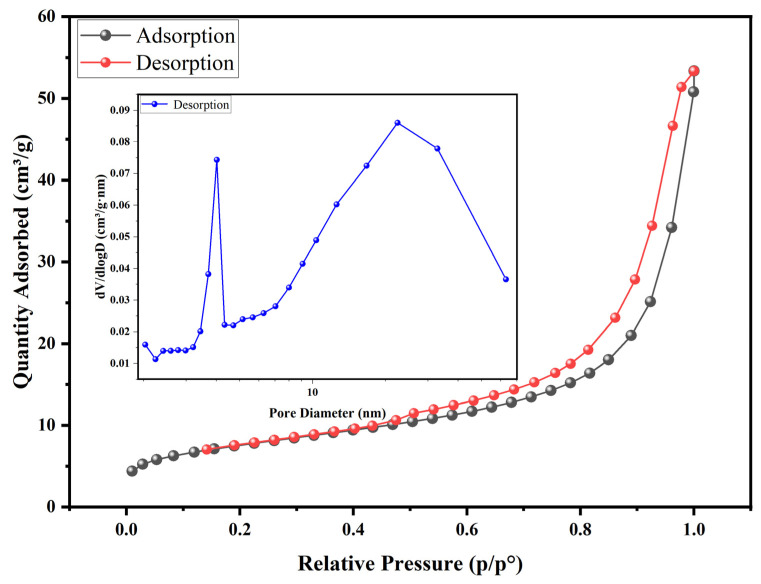
N_2_ adsorption-desorption and pore diameter diagrams.

The BET results are shown in Figure 7. The N_2_ adsorption/desorption isotherm in the figure corresponds to the type IV isotherm in the Brunauer classification, indicating that SA@RM is a typical mesoporous material. Meanwhile, its hysteresis loop belongs to the H3 type, indicating that it is a slit pore or lamellar particle accumulation. The specific surface area, average pore diameter, and pore volume were calculated using isotherm plots and were 26.22 m^2^·g^−1^, 11.97 nm, and 0.0786 cm^3^·g^−1^, respectively, and they were compared with the references as shown in Table 4. The mesoporous dominant structure of SA@RM, with a larger pore volume and a higher specific surface area, provides the fundamental mass transfer channels and active sites for its fluorine adsorption.

**Figure 8:**
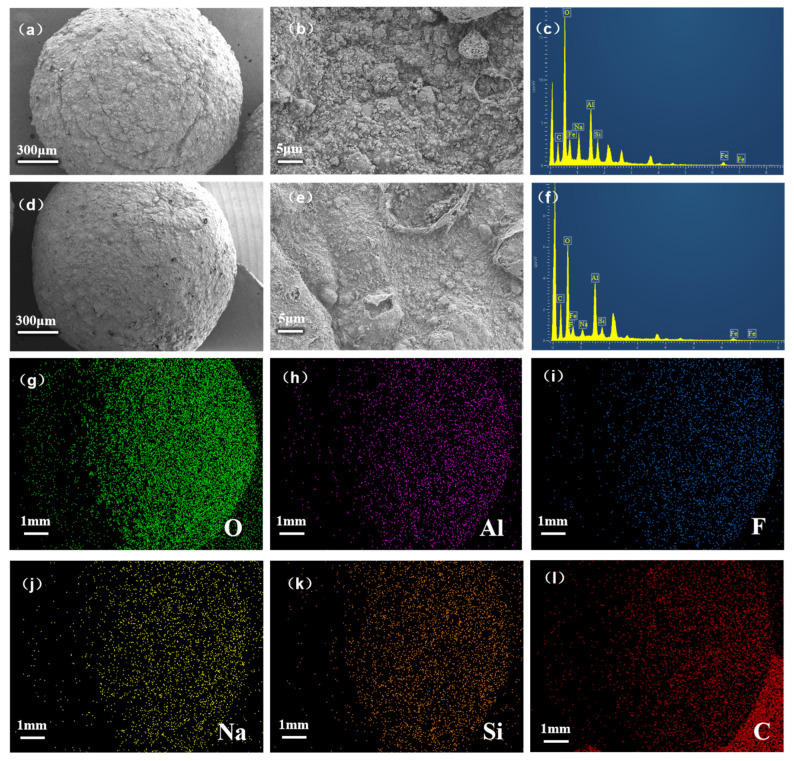
SEM (**a**,**b**,**d**,**e**) and EDS (**c**,**f**–**l**) images of SA@RM before and after fluoride adsorption.

SEM-EDS results of SA@RM before and after adsorption are given (Figure 8) to illustrate the adsorption mechanism.

From the SEM images before adsorption (Figure 8a,b), it can be seen that the surface of SA@RM is rough and uneven with a large specific surface area, which can provide abundant active sites for F^−^ adsorption. After adsorption (Figure 8d,e), the surface of SA@RM was flatter and smoother with significantly decreased adsorption sites. This may be due to the dissolution–reprecipitation of the amorphous bauxite phase under acidic conditions (pH 5), where fluorine-induced surface re-polymerization reduces the roughness: the formation of stable Al-F complexes through ligand exchange (Al-OH + F^−^ → Al-F + OH^−^) permanently occupies aluminum-active sites. Meanwhile, precipitated fluoride salts may also mechanically block pore entrances [30]. EDS analysis confirmed that the main metal element on the adsorbent surface is Al, and it is evenly distributed, which provides the prerequisite for the complexation of Al with F. From the EDS results, it can be seen that the O element content on the surface of SA@RM before adsorption (Figure 8c) is high, while the O element content on the surface of SA@RM after adsorption (Figure 8f) is significantly reduced. At the same time, the absorption peak of the F element appeared. This indicates during the binding of F^−^ to the adsorption sites of SA@RM, there is a ligand exchange between -OH and F^−^, which significantly reduces the O element content. Figure 8i shows the distribution of the F element after adsorption, from which it can be clearly seen that there is F on the surface of SA@RM after adsorption, confirming that F has been adsorbed successfully. At the same time, by comparing Figure 8h,i, it can be observed that the distribution of F highly overlaps with the distribution of Al, further verifying the complexation interaction between Al and F on the SA@RM surface.

#### 3.5.2. FTIR and XPS Analysis

To further illustrate the adsorption mechanism, FTIR and XPS of SA@RM before and after adsorption were analyzed.

**Figure 9:**
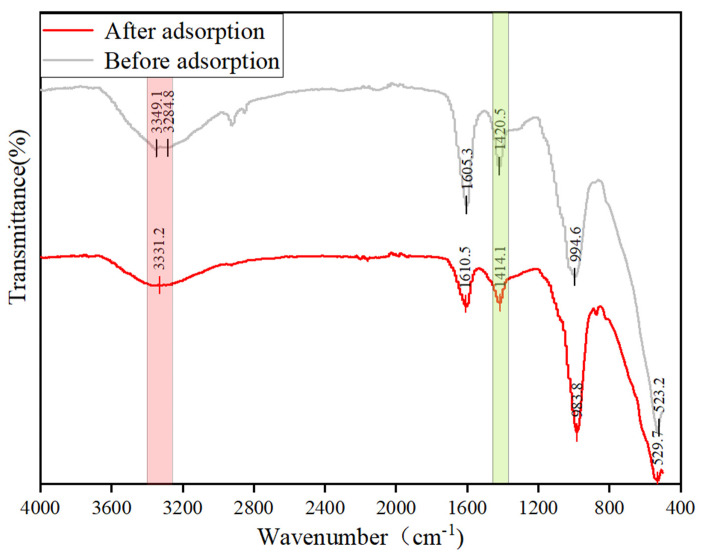
FT-IR patterns of SA@RM before and after fluoride adsorption.

From the FT-IR spectra before and after adsorption (Figure 9), it can be seen that the bands near 3500–3200 cm^−1^ and 1600 cm^−1^ can be attributed to the stretching vibration and deformation vibration of -OH, respectively. Moreover, the intensity of the -OH peaks decrease after adsorption, which further confirms that the ligand exchange between -OH and F^−^ was present in the adsorption process. The absorption peaks at 2900 cm^−1^ are attributed to the stretching vibration of C-H, and the decrease in the absorption peaks after adsorption indicates that there is a decrease in surface-active substances [31]. The absorption peaks at 1500–500 cm^−1^ can be attributed to the deformation vibrations of M-O and M-OH. The peak at 1420 cm^−1^ can be attributed to the characteristic absorption of Al-OH. After adsorption, this peak is also reduced, further confirming the ligand exchange between F and -OH, as well as the complexation with Al. This is consistent with the characterization results of SEM-EDS. The strong absorption peak near 530 cm^−1^ can be attributed to the Al-O bond in the adsorbent, and the peak at 994 cm^−1^ is attributed to the vibration of Fe-O [32]. These two peaks show almost no change before and after adsorption, indicating that M-O-M’s contribution to fluoride removal is negligible.

**Figure 10:**
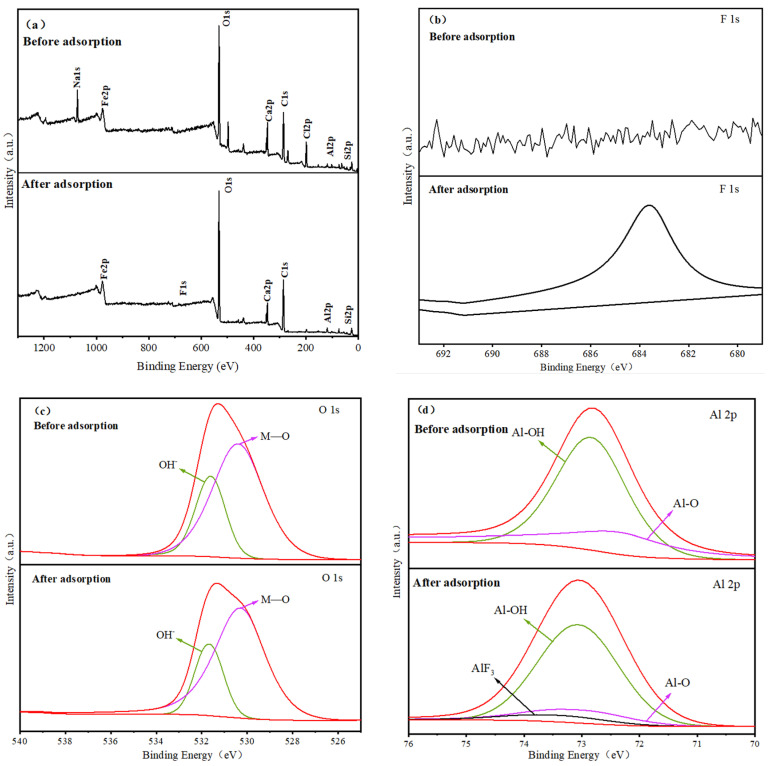
XPS (**a**), F 1s (**b**), O 1s (**c**), and Al 2p (**d**) profiles of SA@RM before and after fluoride adsorption.

As can be seen in Figure 10a, the XPS survey spectra of the material show the presence of a Cl 2p peak at 199.08 eV before adsorption, but the peak disappears after adsorption, while a new peak attributed to F 1s appears at 687 eV (Figure 10b). This indicates that Cl may also participate in the adsorption process of fluoride through ion exchange. The O1s spectra (Figure 10c) show that the peaks of -OH and M-O appear at 531.65 eV and 530.28 eV, respectively. The peak intensities of adsorbed -OH and M-O decreased by 21.8% and 20.8% after adsorption, respectively. This once again demonstrates the ligand exchange interaction between -OH and F. Meanwhile, the Al 2p spectra show (Figure 10d) that a new AlF_3_ peak appears at 73.5 eV after adsorption, the peak locations of Al-OH and Al-O change slightly, and the peak intensities are also reduced by 11.23% and 5.18%, respectively. These changes once again demonstrate the complexation of Al with F. Therefore, Cl, Al, and -OH groups play an important role in the removal of fluoride ions. The above results demonstrate that the adsorption mechanism includes complexation and ion exchange [33]. The whole reaction process can be described by the following equation:

R_n_-Al-Cl_3-n_ + OH^−^→R_n_-Al-(OH)_3-n_ + Cl^−^ (9)

R-Al-X_2_ + F^−^→R-Al-XF + X^−^ (10)

R-Al-X_2_ + 2F^−^→R-Al-F_2_ + 2X^−^ (11)

R_2_-Al-X + F^−^→R_2_-Al-F + X^−^ (12)

n = 1 or 2; X = Cl or OH.

In summary, this adsorption process is characterized by the presence of Cl^−^ or OH^−^ undergoing ion exchange with F^−^ in solution.

## 4. Conclusions

In this research, a reusable adsorbent was synthesized using red mud and sodium alginate as raw materials for the adsorption of low-concentration fluoride wastewater. The adsorbent demonstrated near-complete solid–liquid separation within 30 s (vs. >30 min for conventional red mud slurries), addressing a critical barrier to practical deployment. Kinetic studies showed that the adsorption process conformed to the pseudo-second-order kinetic model, indicating that the adsorption process is more inclined to chemisorption. Meanwhile, the adsorption isotherm fitted the Langmuir adsorption isotherm model also indicates that the adsorption process may involve a chemical reaction. The characterization results of SEM-EDS, FT-IR, and XPS corroborate each other that the adsorption mechanism exists as a ligand exchange of F with -OH, ion exchange of F^−^ with Cl^−^, and its complexation with Al. Compared to other adsorbents, the production cost of SA@RM is extremely low because its raw material is a type of solid waste. Since the red mud is embedded in sodium alginate, it takes advantage of the inherent aluminum reactivity of red mud while enhancing its structural stability, simultaneously valorizing industrial solid waste and enabling efficient water purification. Therefore, it has environmental and economic benefits, making it a promising adsorbent for application.

## Figures and Tables

**Table 1:** Main chemical composition and mass percent of red mud from raw materials.

SiO_2_	Fe_2_O_3_	Al_2_O_3_	CaO and MgO	Na_2_O	K_2_O	TiO_2_	Ignition Loss
16.3%	30.1%	26.25%	1.81%	16.2%	0.128%	3.619%	13.48%

**Table 2:** Kinetic model fitting parameters.

pseudo-first-order kinetic model	q_e_ (mg·g^−1^)	k_1_ (min^−1^)	**R^2^**
$q_{t}=q_{e}(1-e^{-k_{1}t}$)	0.4649	0.0624	0.9423
pseudo-second-order kinetic model	q_e_ (mg·g^−1^)	k_2_ (g·mg^−1^·min^−1^)	R^2^
q_t_ = k_2_q_e_^2^t/(1 + kq_e_t)	0.5341	0.1523	0.9745

**Table 3:** Adsorption isothermal model parameters.

Langmuir adsorption isothermal model	q_max_ (mg·g^−1^)	K_L_ (L·mg^−1^)	R^2^
q_e_ = K_L_q_max_Ce/(1 + Ce)	0.73	0.3843	0.9942
Freundlich adsorption isothermal model	K_F_ (L·mg^−1^)	n	R^2^
q_e_ = K_F_Ce^(-n)^	0.2574	−0.3640	0.9258

**Table 4:** Comparison of BET-related data.

Absorbent	Specific Surface Area (m^2^·g^−1^)	Average Pore Diameter (nm)	Pore Volume (cm^3^·g^−1^)	References
SA@RM	26.22	11.97	0.0786	This study
biosorbent beads	2.74	11.77	0.008	Mirzaei et al. [28]
silica particles	2.201	4.07	0.0022	Zare et al. [29]

## Data Availability

The original contributions presented in this study are included in the article. Further inquiries can be directed to the corresponding author(s).
